# The early responses of VEGF and its receptors during acute lung injury: implication of VEGF in alveolar epithelial cell survival

**DOI:** 10.1186/cc5042

**Published:** 2006-09-13

**Authors:** Marco Mura, Bing Han, Cristiano F Andrade, Rashmi Seth, David Hwang, Thomas K Waddell, Shaf Keshavjee, Mingyao Liu

**Affiliations:** 1 Thoracic Surgery Research Laboratories, Toronto General Research Institute, University Health Network; Department of Surgery, Faculty of Medicine, University of Toronto, 200 Elizabeth Street, Toronto, Canada M5G 2C4

## Abstract

**Introduction:**

The function of the vascular endothelial growth factor (VEGF) system in acute lung injury (ALI) is controversial. We hypothesized that the role of VEGF in ALI may depend upon the stages of pathogenesis of ALI.

**Methods:**

To determine the responses of VEGF and its receptors during the early onset of ALI, C57BL6 mice were subjected to intestinal ischemia or sham operation for 30 minutes followed by intestinal ischemia-reperfusion (IIR) for four hours under low tidal volume ventilation with 100% oxygen. The severity of lung injury, expression of VEGF and its receptors were assessed. To further determine the role of VEGF and its type I receptor in lung epithelial cell survival, human lung epithelial A549 cells were treated with small interference RNA (siRNA) to selectively silence related genes.

**Results:**

IIR-induced ALI featured interstitial inflammation, enhancement of pulmonary vascular permeability, increase of total cells and neutrophils in the bronchoalveolar lavage (BAL), and alveolar epithelial cell death. In the BAL, VEGF was significantly increased in both sham and IIR groups, while the VEGF and VEGF receptor (VEGFR)-1 in the lung tissues were significantly reduced in these two groups. The increase of VEGF in the BAL was correlated with the total protein concentration and cell count. Significant negative correlations were observed between the number of VEGF or VEGFR-1 positive cells, and epithelial cells undergoing cell death. When human lung epithelial A549 cells were pre-treated with 50 nM of siRNA either against VEGF or VEGFR-1 for 24 hours, reduced VEGF and VEGFR-1 levels were associated with reduced cell viability.

**Conclusion:**

These results suggest that VEGF may have dual roles in ALI: early release of VEGF may increase pulmonary vascular permeability; reduced expression of VEGF and VEGFR-1 in lung tissue may contribute to the death of alveolar epithelial cells.

## Introduction

Acute lung injury (ALI) along with its severe form, acute respiratory distress syndrome (ARDS), is one of the most challenging conditions in critical care medicine. ALI/ARDS can result from a direct insult in the lung or an indirect insult from other organs mediated through the systemic circulation [1,2]. ARDS of both etiologies results in acute inflammatory responses leading to lung dysfunction [3]. Mesenteric ischemia-reperfusion represents an important cause of extrapulmonary ARDS, as gut mucosal perfusion deficits appear to be instrumental in the propagation of multiple organ failure, of which the most vulnerable organ is the lung [4].

Increased pulmonary permeability that leads to diffuse interstitial and pulmonary edema is one of the most important manifestations of ALI/ARDS [5]. Increased cell death has been proposed to be an important component for lung tissue damage [6]. Vascular endothelial growth factor (VEGF) and its receptors are critical in the regulation of both vascular permeability and endothelial cell survival. Therefore, VEGF and related molecules may have important roles in the development of ALI/ARDS [7].

The VEGF system consists of several VEGF isoforms and VEGF receptors (VEGFRs). Most studies have focused on VEGF-A (from hereon the abbreviation VEGF refers to VEGF-A) because it plays an essential role in angiogenesis and vascular permeability [8,9]. In the lung tissue, VEGF is highly compartmentalized and mainly produced in epithelial cells, whereas endothelial cells are suggested as its major target [10,11]. Most of the angiogenic activities of VEGF as well as its effects on vascular permeability are mediated by its receptor Flk-1 (VEGFR-2) [12], while the functions of Flt-1 (VEGFR-1), especially its role in ALI, are largely unknown.

Pulmonary permeability is controlled by both endothelial and epithelial layers. Pulmonary injury in ARDS causes widespread destruction on both sides of the epithelial-endothelial barrier [5,13]. The effect of VEGF on endothelial cell permeability and survival has been demonstrated in both *in vitro *and *in vivo *studies [14,15]. The effect of the VEGF system on the integrity of pulmonary epithelium is unclear.

VEGF may contribute to the development of noncardiogenic pulmonary edema in ALI/ARDS [16]. Systemic overexpression of VEGF has been shown to cause widespread capillary leakage in multiple organs [9], and high plasma levels of VEGF were found in ARDS patients [16]. However, studies from animal models as well as from ARDS patients have shown that decreased levels of VEGF in the lung are associated with a worse prognosis [17-19]. Therefore, the role of VEGF and related molecules in ALI/ARDS is controversial [7]. One possible explanation is that VEGF may play different roles at different stages of the development of and recovery from ALI/ARDS [7]. We hypothesized that, in the early stage of lung injury, the release of VEGF from alveolar epithelial cells and leukocytes induced by acute inflammatory response may increase the vascular permeability and contribute to the formation of interstitial edema in the lung, whereas reduced VEGF and its receptors in alveolar epithelial cells due to tissue damage may lead to cell death. In the present study, we investigated the release of VEGF, and the expression and distribution of VEGF and its receptors in the lung during the early onset of ALI induced by intestinal ischemia-reperfusion (IIR), a well-established model of extrapulmonary ARDS [20,21]. Since expression levels of VEGF and VEGFR-1 were negatively correlated with alveolar epithelial cell death, we investigated the potential roles of these two proteins on epithelial survival by reducing their expression with small interference RNA (siRNA) in A549 cells, a human lung epithelial cell line with partial type II pneumocyte characteristics.

## Materials and methods

### Intestinal ischemia-reperfusion model in mice

We randomized 6 to 9 week old male C57BL6 mice (weight = 25.8 ± 2.7 g) into IIR, sham (sham-operated), or control groups. The animals subjected to IIR or sham operation were anesthetized with an intraperitoneal injection of acepromazine (10 mg/ml)-ketamine (100 mg/ml) (10:1, 0.15 ml). Tracheostomy was performed after blunt dissection of the neck and exposure of the trachea. A metal cannula for mouse (1.0 mm; Harvard Apparatus, St Laurent, Canada) was inserted into the trachea, and animals were connected to a volume-controlled constant flow ventilator (Inspira Advanced Safety Ventilator, Harvard Apparatus). Anesthesia was continuously maintained with isoflurane and body temperature was maintained at 37°C by an immersion thermostat throughout the experiment. In the IIR group the abdomen was rinsed with betadine, a lower midline laparatomy was performed and the superior mesenteric artery was identified and occluded below the celiac trunk with an arterial microclamp. Intestinal ischemia was confirmed by paleness of the jejunum and ileum. After 30 minutes the clamp was removed, 0.5 ml of sterile saline at 37°C was injected into the peritoneal cavity and the skin was sutured. The same procedures were carried out in the sham group, but the mesenteric artery was not clamped. The animals were then ventilated for four hours with a tidal volume of 6 ml/kg, inspiratory oxygen fraction 1.0, inspiratory/expiratory ratio 1:2 and a frequency of 140 breaths per minute. An esophageal catheter (Harvard Apparatus) was applied to eight animals per group for measurement of dynamic lung compliance. The left femoral artery was cannulated in four animals per group for measurement of mean arterial blood pressure. Airways pressures, dynamic lung compliance and blood pressure were continuously monitored throughout the four hour period of mechanical ventilation (MV) with HSE-USB acquisition hardware and Pulmodyn software (H Sachs Elektronik, March-Hugstetten, Germany). The control group consisted of mice spontaneously breathing room air. The experimental protocol was approved by the Toronto General Hospital Animal Care and Use Committee. All mice received care in compliance with the Principles of Laboratory Animal Care formulated by the National Society for Medical Research, and the Guide for the Care and Use of Experimental Animals formulated by the Canadian Council on Animal Care.

All animals were sacrificed by exsanguinations. The lungs were sub-grouped either for histological evaluation and immunohistochemistry (*n *= 4/group) or bronchoalveolar lavage (BAL; *n *= 12/group). Blood samples were collected (*n *= 8 animals/group) at the end of the experiment by puncture of the aorta. After centrifugation at 4,000 g for 10 minutes, plasma samples were stored at -20°C before use.

### Assessment of acute lung injury

Lungs for histological evaluation were removed *en bloc *and inflated at a 20 cm height with 4% paraformaldehyde in PBS for fixation. Sections (4 μm) were either stained with haematoxylin and eosin or processed for immunohistochemistry. A pulmonary pathologist performed the histological analysis in a blinded fashion. The degree of lung injury was determined using the grading system developed by Ginsberg and colleagues [22].

BAL was performed by instilling 0.5 ml of saline through the endotracheal tube and gently aspirating back. This was repeated twice and the amount of fluid recovered was recorded. An aliquot of BAL fluid (50 μl) was diluted 1:1 with trypan blue for total cell counting using a haemocytometer. In 8 animals per group, an aliquot of BAL fluid (80 μl) underwent cytospin (72 g, 5 minutes) and the cells collected were stained using the Harleco Hemacolor staining kit (EMD Science, Gibbstown, NJ, USA). Differential cell count was conducted by counting of at least 500 cells. The remainder of the lavage fluid was centrifuged (4,000 g, 10 minutes), and the supernatant was stored at -20°C until measurement of protein concentration with Bradford assay (Bio-Rad Laboratories, Hercules, CA, USA).

For the Evans Blue Dye (EBD) permeability assay, the left jugular vein was isolated and cannulated in four animals per group. An EBD solution (5 mg/ml) was injected into the left jugular vein (30 mg/kg) 30 minutes prior to sacrifice of the animal. The BAL fluid and plasma were collected and the optical density of EBD was read at 630 nm with a spectrophotometer (Opsys MR, Thermo Labsystems, Franklin, MA, USA). The optical density ratio of BAL/plasma EBD was then calculated.

### Enzyme-linked immunosorbent assay

VEGF levels were determined in the BAL supernatants and plasma samples using an ELISA kit (DuoSet Mouse VEGF, R&D Systems, Minneapolis, MN, USA) that recognizes VEGF isoforms with either 120 or 164 amino acids. Assays were performed in duplicate following the manufacturer's instructions.

### Immunohistochemistry

For immunohistochemistry (IHC), lung tissue slides (4 μm) were pre-treated with 0.25% Triton X-100 for five minutes and blocked for endogenous peroxidase and biotin with 0.3% H_2_O_2 _in methanol. The slides were incubated with designated primary antibodies, with a dilution of 1:200 for VEGF (sc-507), 1:20 for VEGFR-1 (sc-316) and VEGFR-2 (sc-505) from Santa Cruz Biotechnology (Santa Cruz, CA, USA), for 32 minutes at 42°C, and then with a secondary antibody (1:600) for 20 minutes. Detection was done by Avidin Biotin Complex system with 3–3 diaminobenzidine as chromogen from a VECTSTAIN ABC kit (PK-4001, Vector Laboratories, Burlingame, CA, USA). Cell nuclei were counterstained with hematoxylin. Non-immune serum instead of the primary antibody was used for negative controls (data not shown). The VEGFR-1 staining was abolished by pre-incubation of slides with a specific blocking peptide (sc-316p, Santa Cruz) (data not shown).

For quantitative analysis, 10 optical fields of alveolar area from each animal (4 mice/group), not including major airways or vessels, were randomly chosen at 1,000 × magnification. The numbers of cells with VEGF, VEGFR-1 or VEGFR-2 positive-staining as well as the total cell nuclei in the chosen fields were counted, respectively, in a double blind fashion. The number of positive-stained cells was expressed as a percentage of the total cells. The staining intensities in bronchial epithelium (ciliated or non-ciliated cells), alveolar epithelium (type I and type II cells), interstitial cells, vascular endothelium and alveolar macrophages were also scored semi-quantitatively [23]. Different cell types were identified by their location and morphology. This screening test could provide an overall impression of the changes of VEGF and its receptors in different cell types.

### TUNEL-cytokeratin double fluorescent staining

Terminal transferase dUTP nick end labeling (TUNEL) staining (*In Situ *Cell Death Detection Kit, TMR Red, Roche, Penzberg, Germany) was used to assess cell death in the lung tissues after deparaffinization, dehydration and permeabilization with 10 μg/ml proteinase K in 10 mM Tris/HCl, pH 7.4–8, for 15 minutes. The slides were then stained for cytokeratin by incubating with an anti-cytokeratin-18 monoclonal antibody (1:25, Chemicon, Temecula, CA, USA) at 4°C overnight, and with a fluorescent-FITC-conjugated goat anti-mouse IgG (1:500, Biotium, Hayward, CA, USA) at room temperature for 1 h. Label solution without terminal transferase for TUNEL or non-immune serum was used as negative controls. Tetramethylrhodamine (TMR)-labeled TUNEL-positive nucleotides and FITC-labeled cytokeratin-positive epithelial cells were detected under fluorescence microscope. Ten fields were randomly chosen from each animal (4 mice/group) at 1,000 × magnification and each field contained approximately the same number of alveoli without major airways or vessels. The number of TUNEL-cytokeratin double positive cells and the total cytokeratin positive cells per optical field were quantified. An epithelial cell death index for each animal was calculated as: (TUNEL-cytokeratin positive cells/cytokeratin positive cells) × 100%.

### Western blotting

The protocols for sample preparation and western blotting of lung tissue lysate have been previously described [24-27]. The protein concentration from homogenized snap-frozen lung samples (four from each group) was determined by the Bradford method. Equal amounts of protein from each sample were boiled in SDS sample buffer and subjected to SDS-PAGE. Proteins were transferred to nitrocellulose membranes. Nonspecific binding was blocked by incubation of membranes with 5% (w/v) nonfat milk in PBS for 60 minutes. Blots were incubated with the designated antibody (VEGF sc-507, VEGFR-1 sc-316, or VEGFR-2 sc-6251 antibodies, Santa Cruz Biotechnology) at 1:1,000 dilution overnight at 4°C. The blots were then washed with PBS-0.05% Tween 20 and incubated for 60 minutes at room temperature with horseradish peroxidase-conjugated goat anti-rabbit (1:30,000 dilution) or anti-mouse (1:20,000 dilution) IgG (both from Amersham, Oakville, Canada). After washing, blots were visualized with an enhanced chemiluminescence detection kit (Amersham). We stripped and reprobed blots with antibody for glyceraldehyde-3-phosphate dehydrogenase (GAPDH) as a housekeeping control. Autoradiographs were quantified using a densitometer (GS-690; Bio-Rad Laboratories) and normalized to the GAPDH control.

### Real-time RT-PCR

Quantitative real-time reverse transcriptase PCR (RT-PCR) analysis of the RNA expression of VEGF, VEGFR-1 and VEGFR-2 was performed on RNA isolated from frozen lung tissues (four animals/group) as previously described [28]. The primer sequences are available upon request.

### VEGF and VEGFR-1 knock-down with siRNA in A549 cells

A549 cells were cultured in DMEM with 10% fetal bovine serum to about 50% confluence in 24-well plates, and then treated with 50 nM of siRNA against either VEGF (M-003550) or VEGFR-1 (M-003136) mRNA, or a non-specific duplex RNA (D-001206-13-05) as negative control (SMARTpool, Upstate, Charlottesville, VA, USA) using oligofectamine as transfection reagent (Invitrogen, Carlsbad, CA, USA). At 24 h after transfection, cell morphology was examined with phase-contrast microscopy, and cell viability was determined with an XTT assay following the manufacturer's instructions (Roche). The knock-down effect at the protein level in the cells was determined by immunofluorescent staining and western blotting with polyclonal antibodies against VEGF or VEGFR-1 (Santa Cruz), respectively. The immunoflurescent staining was visualized with a TMR-conjugated anti-rabbit IgG (1:400) as the secondary antibody. The protocol for immunofluorescent staining has been previously described in detail [28-30].

### Statistical analyses

All data are expressed as mean ± standard deviation and were analyzed with JMP software (SAS Institute, Cary, NC, USA). Distribution analysis was performed to test skewing for all variables. The non-parametric Kruskal-Wallis (two-tailed) test was used for comparison of multiple groups, followed by the Dunn's test for comparisons between individual groups. Correlation studies were performed with Spearman rank correlation (*Rho factor*). *P *values less than 0.05 are regarded as significant.

## Results

### Intestinal ischemia-reperfusion-induced acute lung injury

Animals in the IIR group developed ALI. The overall survival was 50% in the IIR group, while no mortality was observed in the sham group within the 4 h experimental period. The distributive shock following the release of proinflammatory mediators from the injured intestine may be the cause of this high rate of mortality, as the blood pressure in the IIR group decreased significantly, in comparison with that in the sham group (Table 1). The mean blood pressure in the sham group was similar to that described in mechanically ventilated C57BL6 mice under anesthesia [31].

The total cell number in the BAL was significantly increased in the IIR group, compared with that in the sham (*p *< 0.05) and control (*p *< 0.01) groups. The differential cell count showed a significantly higher percentage of neutrophils in the IIR group. A significant increase of total cell counts and protein concentration was also observed in the BAL from the sham group compared to that of control animals, which may be due to the high concentration of oxygen used for ventilation. However, when EBD assay was used to further assess the pulmonary permeability, a significant increase in the BAL/plasma EBD ratio was only detected in the IIR group (Table 1). After 4 h of reperfusion, the lung compliance did not significantly change in the sham group, whereas it was significantly decreased in the IIR group, in comparison with the basal line (*p *< 0.05; Table 1).

**Figure 1:**
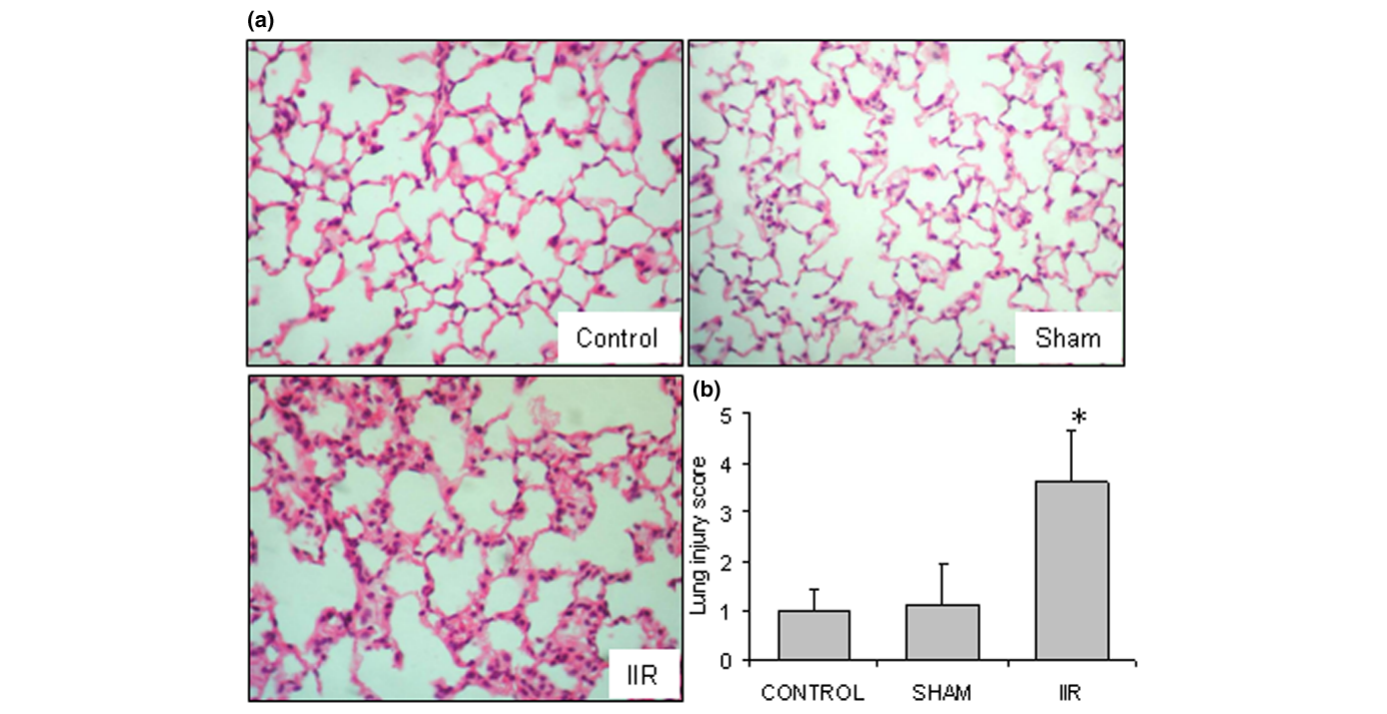
Intestinal ischemia reperfusion (IIR)-induced acute lung injury. **(a) **In comparison with control group, lung histology (haematoxylin and eosin, magnification 400×) shows a minor infiltration of leukocytes in the sham group. In the IIR group, a diffuse increase of interstitial cellularity, with both mononuclear cells and neutrophil infiltration, interstitial edema, and vascular congestion were observed. Slides shown are representatives of four animals from each group. **(b) **The severity of lung tissue injury in each group was quantitatively scored; *p < 0.05 versus control and sham groups.

The histological studies showed a minimal and focused increase of interstitial cellularity in the sham group and a diffuse increase, due to infiltration of both mononuclear cells and neutrophils, in the IIR group (Figure 1a). Diffuse interstitial edema and vascular congestion were observed in the IIR group (Figure 1a). These features are compatible with those observed in early extrapulmonary ARDS [3]. As a result, the lung injury score was significantly increased in the IIR group (Figure 1b).

### VEGF increased in the BAL but decreased in the lung tissues after IIR

**Figure 2:**
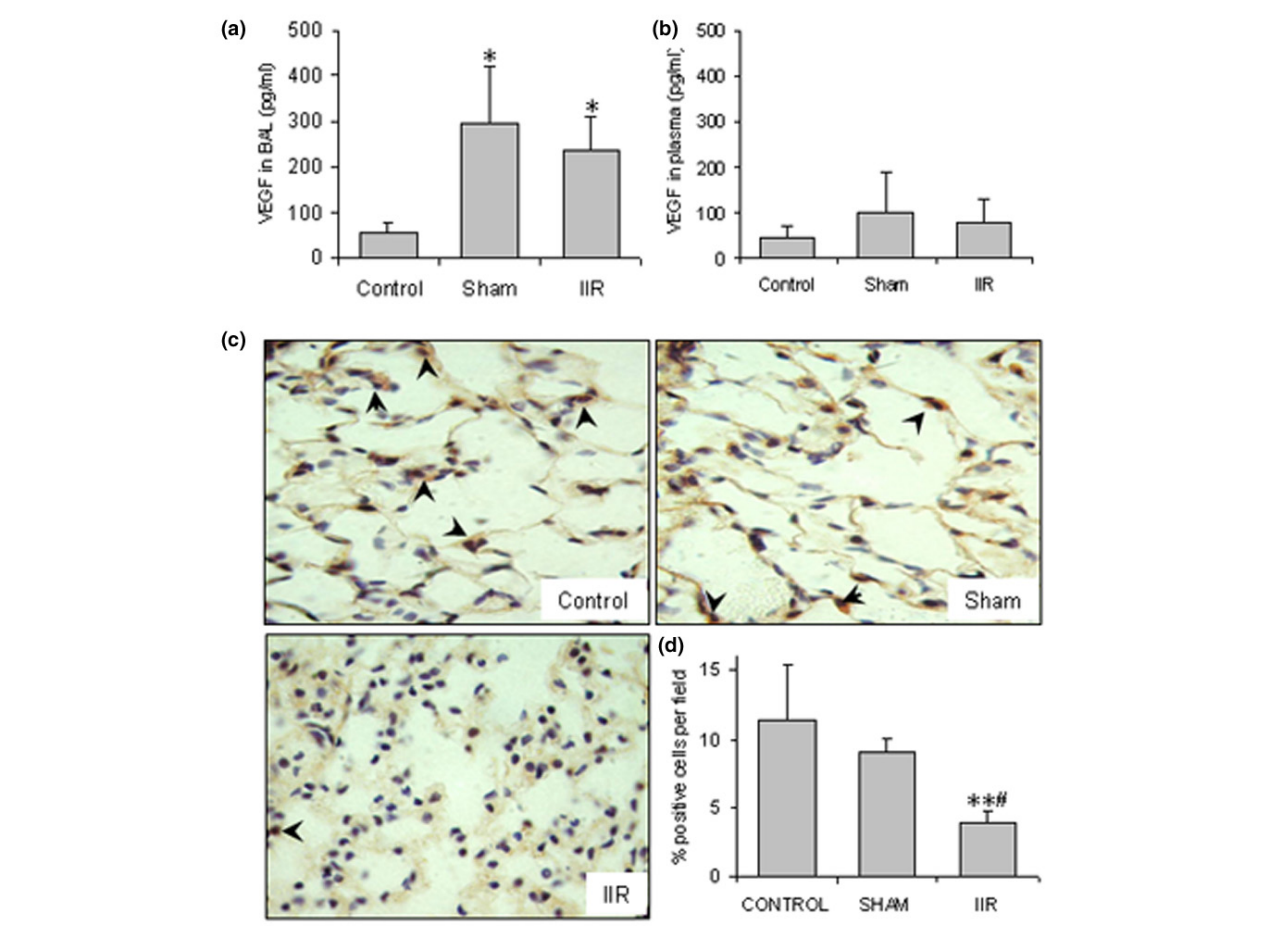
Intestinal ischemia reperfusion (IIR)-induced changes in vascular endothelial growth factor (VEGF) expression in the lung. **(a) **VEGF in the bronchoalveolar lavage (BAL) fluid (*n *= 12/group); *p < 0.05 compared with the control. **(b) **VEGF in the plasma (*n *= 8/group). **(c) **VEGF immunostaining in the lung tissues (*n *= 4/group). Slides shown are representatives for each group (magnification 1,000×), and arrowheads indicate the examples of positive stained cells (in brown). **(d) **Quantification of VEGF positive cells per field. Ten fields were counted from each animal and four animals from each group. In the IIR group, the number and intensity of positive stained cells in the alveolar walls were remarkably decreased. **p < 0.01 compared with the control group; ^#^*p *< 0.05 compared with the sham group.

We then investigated the alterations of VEGF and its receptors in IIR-induced ALI. A significant increase of VEGF in the BAL fluid was found from both the sham and IIR groups (Figure 2a), while no difference in the plasma levels of VEGF was found among the groups (Figure 2b).

The expression and distribution of VEGF in the lung tissues were examined with IHC. VEGF expression in control and sham-operated animals was characterized by strong staining of bronchial epithelial cells and moderate to diffuse staining of vascular endothelial cells (Table 2). Type II epithelial cells and occasional alveolar macrophages were VEGF-positive (Figure 2c, Table 2). In the IIR group, the intensity and the number of VEGF-positive cells were clearly decreased, especially in alveolar epithelial cells and bronchial epithelial cells (Figure 2c, Table 2). The percentage of identifiable VEGF-positive cells in the alveolar walls was quantified in a double-blinded fashion and expressed as a percentage of the total number of cells in each field, which was significantly lower in the IIR group (Figure 2d).

### Decreased VEGFR-1 expression in the injured lung tissues

**Figure 3:**
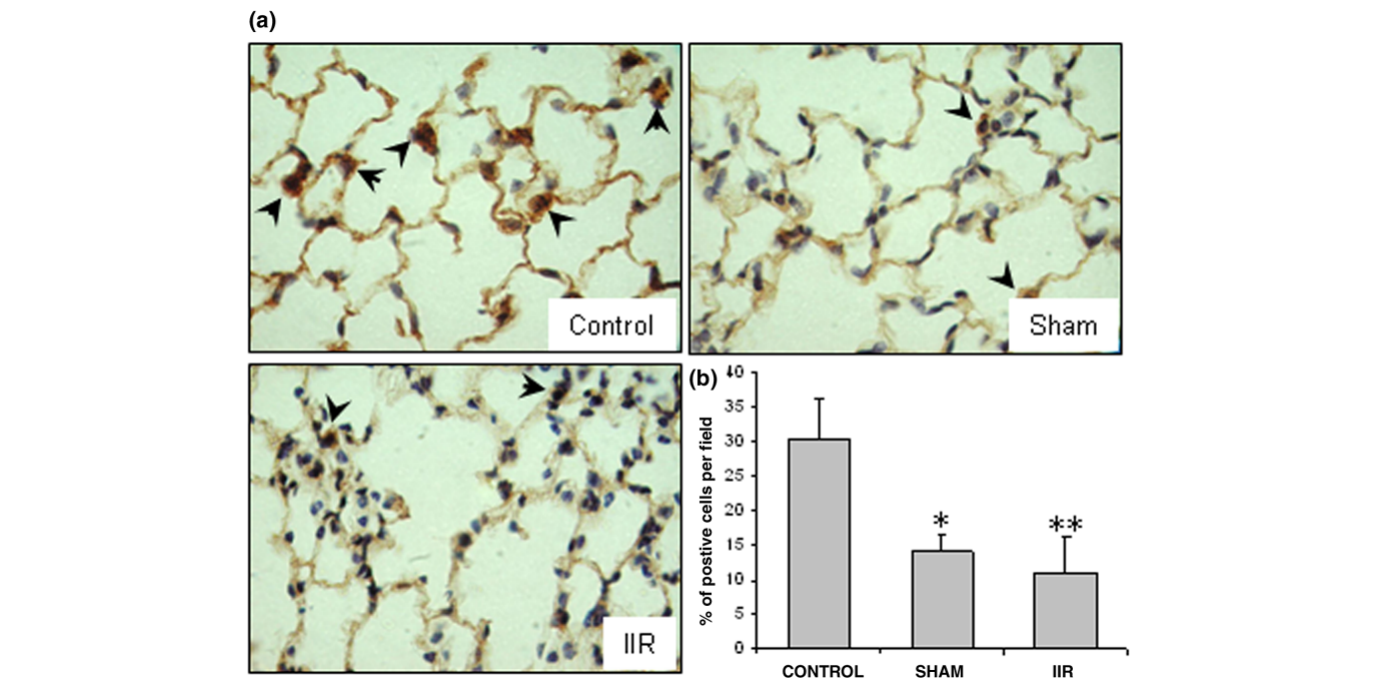
Reduced vascular endothelial growth factor receptor (VEGFR)-1 expression in the lung tissue. **(a) **VEGFR-1 immunostaining (*n *= 4/group). Slides shown are representatives of each group (magnification 1,000×). Positively stained cells are in brown (examples are shown with arrowheads). **(b) **Quantification of VEGFR-1 positive cells per field. Ten fields were counted from each animal and four animals from each group. The number of positive staining cells in the alveolar walls was decreased in the sham group, and further reduced in the intestinal ischemia reperfusion (IIR) group. *p < 0.05 and **p < 0.01 compared with the control group.

Strong staining of both airway and alveolar epithelial cells characterized the VEGFR-1 distribution in the control group (Table 2, Figure 3a). In the sham group, a similar staining pattern was observed, with much fewer positive cells in the alveolar units (*p *< 0.05). The decrease of VEGFR-1 positive cells was more significant in the IIR group (*p *< 0.01), due to the reduced staining on type I epithelial cells, interstitial cells and alveolar macrophages (Figure 3, Table 2).

### Redistributed VEGFR-2 expression in the injured lung tissues

**Figure 4:**
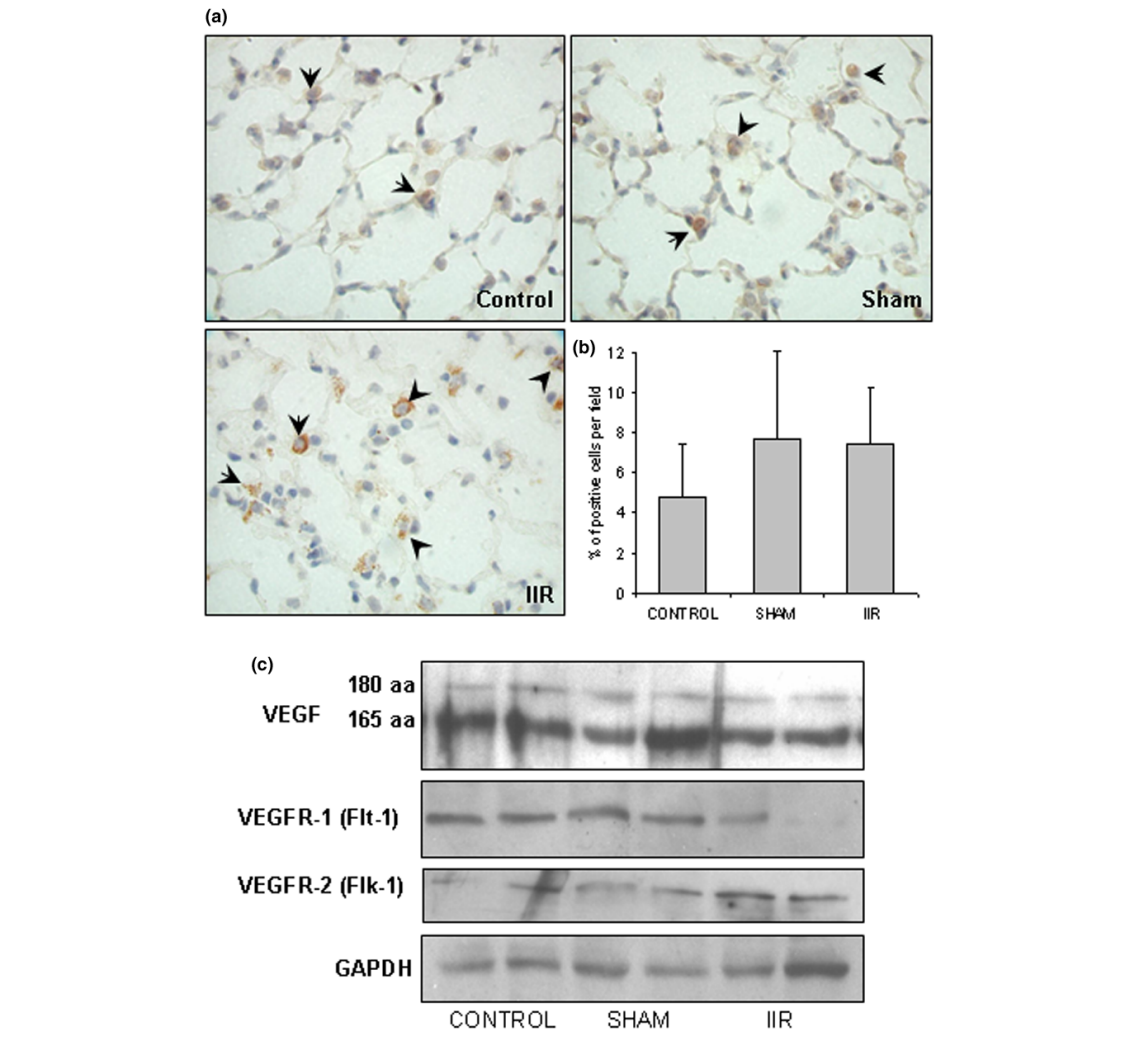
Intestinal ischemia reperfusion (IIR)-induced changes of vascular endothelial growth factor receptor (VEGFR)-2 in the lung tissue and immunoblotting of VEGF and its receptors. **(a) **VEGFR-2 immunostaining (four animals per group). Slides (magnification 1,000×) shown are representatives of indicated groups. Positively stained cells are in brown (examples are indicated with arrowheads). In the IIR group, some of the positive cells appear to be interstitial monocytes with strong staining in the cytoplasm. **(b) **Quantification of VEGFR-2-positive cells per field. Ten fields were counted from each animal and four animals from each group. **(c) **Western blotting for VEGF and its receptors. Results from two animals per group are used as examples. The optical density of blot bands were quantified with desitometry and normalized to that of glyceraldehyde-3-phosphate dehydrogenase (GAPDH) as controls. No significant difference was found among these three groups.

The VEGFR-2 immunostaining in control and sham groups revealed strong staining on bronchial epithelial cells (Table 2), with occasional and weak staining of alveolar type II epithelial cells, macrophages and vascular endothelial cells (Figure 4a). In the IIR group, the staining was redistributed in the cytoplasm, with a granular-like appearance in the infiltrated mononuclear cells in the interstitium. An increased number of positively stained type II cells was also observed (Figure 4a). However, the total number of positively stained cells in the alveolar units did not significantly change (Figure 4b). No change was observed in the bronchial epithelium and vascular endothelium (Table 2).

We used western blotting to determine the protein levels of VEGF and its receptors in lung tissue. Results from two animals are shown in Figure 4c as examples. When quantified with densitometry, no statistical significance was found. We also used real-time quantitative RT-PCR to measure the mRNA levels of VEGF and its receptors. The results are also not statistically significant (data not shown).

### Correlations of VEGF and its receptors with lung injury and epithelial cell death

We then examined whether the VEGF concentration in the BAL correlates with parameters related to vascular permeability (Table 3). The VEGF level was significantly correlated with the total protein concentration in the BAL fluid. A significant correlation was also found between VEGF concentration and total cell count. The differential cell counting further revealed that VEGF was correlated positively with the percentage of macrophages, but inversely with neutrophils (both percentage and cell number) in the BAL (Table 3).

**Figure 5:**
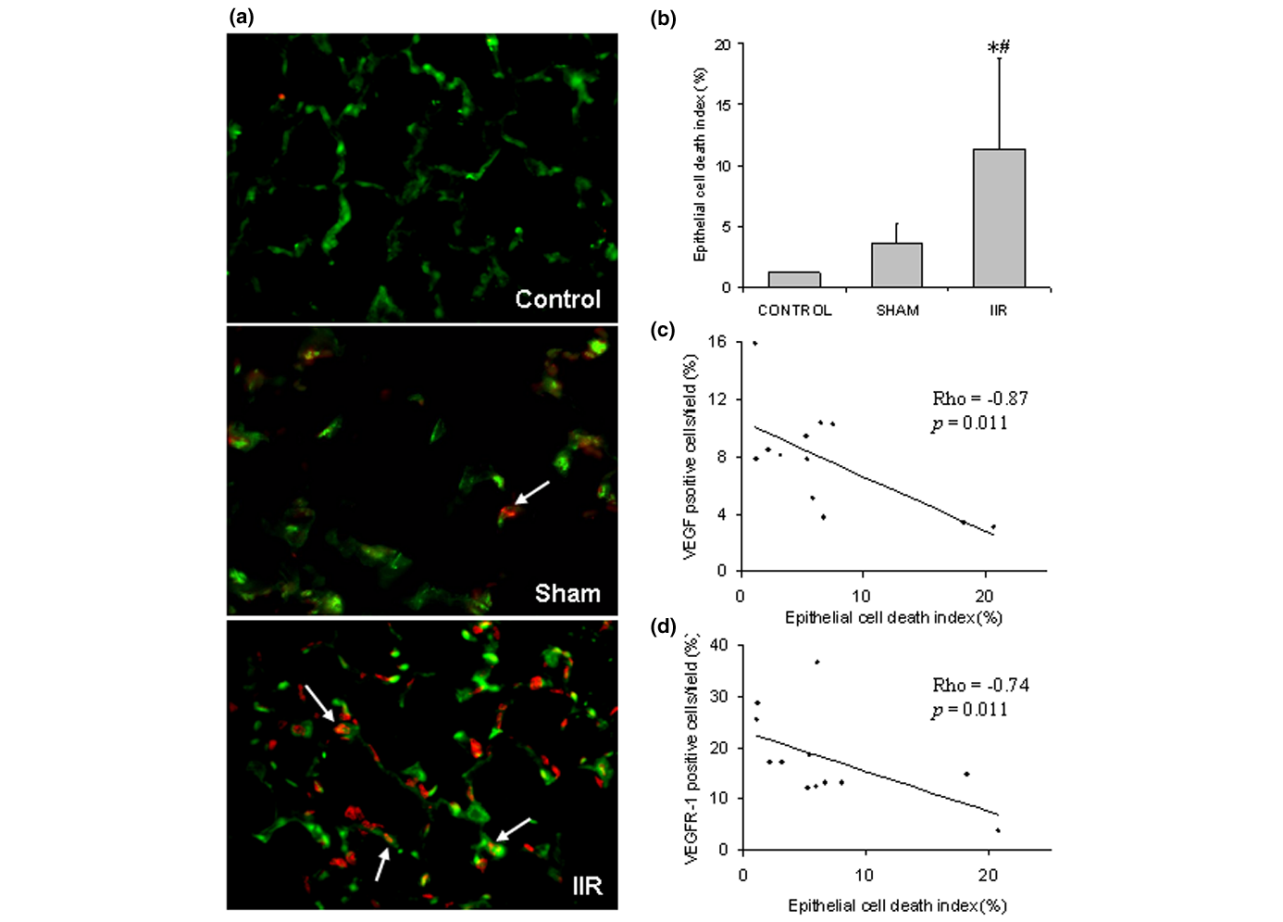
Intestinal ischemia reperfusion (IIR)-induced alveolar epithelial cell death is negatively correlated with vascular endothelial growth factor (VEGF) and vascular endothelial growth factor receptor (VEGFR)-1 expression. **(a) **Double fluorescents staining TUNEL (red)-cytokeratin (green). An increased number of epithelial cells (in green) undergoing cell death (in red) was detected in the IIR group. Slides shown are representatives of four animals from each group. **(b) **Epithelial cell death index was quantified by TUNEL-cytokeratin double positive cells over cytokeratin positive cells of each field (ten fields were quantified from each animal); *p < 0.05 compared with the control group; ^#^*p *< 0.05 compared with the sham group. **(c) **Relationship between TUNEL-positive-epithelial cells and VEGF-positive cells. **(d) **Relationship between TUNEL-positive-epithelial cells and VEGFR-1-positive cells.

To verify if epithelial cell death was related to the down-regulation of VEGF and VEGFR-1, TUNEL and cytokeratin double staining was performed. An increased number of TUNEL-cytokeratin double positive cells was found in the IIR group (Figure 5a), which was statistically significant when quantified and compared with the cell death in the control and sham groups (Figure 5b). The number of VEGF positive cells in the alveolar units was negatively correlated with the number of TUNEL-cytokeratin positive cells (*Rho *= -0.87, *p *= 0.001; Figure 5c). Similarly, the number of VEGFR-1 positive cells was negatively correlated with that of TUNEL-cytokeratin positive cells (*Rho *= -0.74, *p *= 0.011; Figure 5d). No significant correlation was found with the number of VEGFR-2 positive cells. These results suggest that reduced VEGF and VEGFR-1 expression in lung tissue may contribute to the death of lung epithelial cells.

### Knock-down of either VEGF or VEGFR-1 reduced lung epithelial cell viability

**Figure 6:**
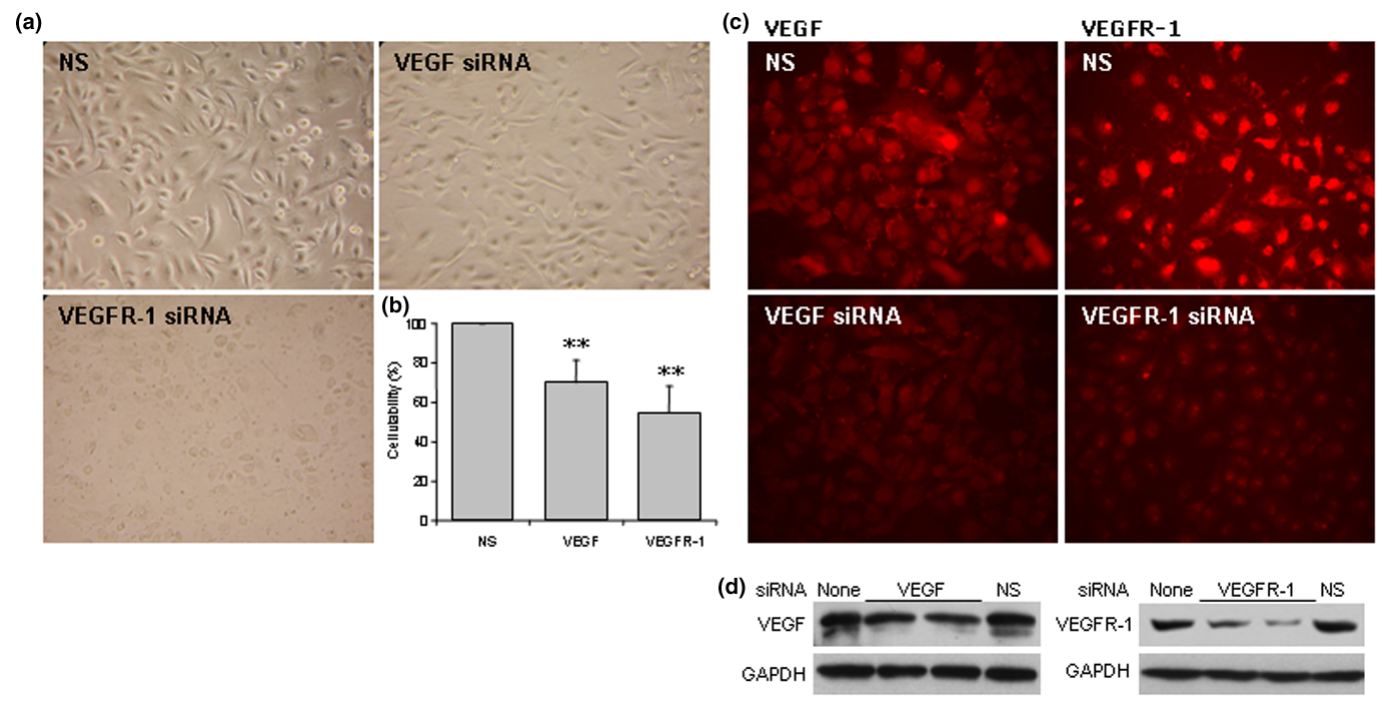
Lung epithelial cell death induced by vascular endothelial growth factor (VEGF) or vascular endothelial growth factor receptor (VEGFR)-1 knock-down. Human A549 cells were cultured in 24-well plates and transfected with 50 nM of small interference RNA (siRNA) specifically against either VEGF or VEGFR-1, or with a non-specific duplex RNA (NS) as control for 24 h. **(a) **VEGF or VEGFR-1 siRNA induced changes in cell number and/or cell morphology in A549 cells. Pictures shown are representatives for each treatment (magnification 400×). **(b) **XTT assay for cell viability. The data shown are mean ± standard deviation from three separated experiments. **p < 0.01 versus the non-specific control. **(c) **VEGF and VEGFR-1 immunofluorescent staining at 24 h after treatment with different RNA duplexes. Decreased staining was noted in siRNA treated cells. **(d) **Western blots confirmed that siRNA specifically reduced VEGF or VEGFR-1 protein expression.

To further determine the roles of VEGF and VEGFR-1 in lung epithelial cell survival, human lung epithelial A549 cells were pre-treated with pooled siRNAs that contain at least four selected siRNA duplexes specifically against either human VEGF or VEGFR-1. The specificity and efficacy of these siRNAs have been characterized by the manufacturer. In comparison with the non-specific duplex RNA control, reduced cell number and changes in cell morphology was observed (Figure 6a), and a significant reduction in cell viability (Figure 6b; *p *< 0.01) was detected by XTT assay at 24 hours after either VEGF or VEGFR-1 siRNA treatment. The protein levels of VEGF or VEGFR-1 were reduced by siRNA treatment, as confirmed by immunofluorescent staining (Figure 6c) and western blotting (Figure 6d) with specific antibodies against VEGF or VEGFR-1, respectively. The non-specific duplex RNA had no effects on cell viability and protein levels of VEGF and VEGFR-1 in comparison with non-treated control.

## Discussion

The present study is a comprehensive report on the early responses of VEGF and its receptors in an animal model of ALI. We observed an increase of VEGF in the BAL, a decreased expression of VEGF and VEGFR-1, and an altered expression pattern of VEGFR-2 in the lung tissue. The VEGF levels in BAL correlated with pulmonary permeability. Decreased expression of VEGF and VEGFR-1 in the lung tissue negatively correlated with death of alveolar epithelial cells. Using cell culture as a model system, we further demonstrated that VEGF and/or VEGFR-1 may play an important role in lung epithelial cell survival.

The VEGF levels in the BAL were increased in both sham and IIR groups, which suggests that these changes may be related to hyperoxia and/or MV, applied to animals in both groups [32-34]. Although it is well known that hypoxia is the most potent regulator of VEGF gene expression and protein production [35], an oxygen-independent up-regulation of VEGF and vascular barrier dysfunction has been observed [36]. A rise in VEGF levels in the BAL in a chronic hyperoxia model in piglets has also been reported [32]. This could be explained at least partially by the release of VEGF from extracellular matrix through hyperoxia-induced proteolytic cleavage [33,34]. Although animals in this study were ventilated with low tidal volume, we cannot exclude the contribution of mechanical factors to the release of VEGF [37], or an addictive effect between MV and hyperoxia.

Alveolar macrophages represent a potential source of VEGF in ALI [16]. We found a positive correlation between the VEGF levels and percentage of macrophages in the BAL. The granules in the neutrophils also contain VEGF and may represent an additional source of VEGF [38]; however, proteases released by these cells may cleave VEGF [39], which could explain the negative correlation between VEGF levels and the number or percentage of neutrophils in the BAL. The numbers of observations in these correlation studies are small; thus, these results should be interpreted with caution.

We noted a significant correlation of the VEGF concentrations with the total protein concentrations and with the total cell counts in the BAL. High concentrations of VEGF within the lung may contribute to the development of pulmonary edema by alternating the state of the adherens junction complexes on the endothelium [40]. An alternative explanation for this correlation is that the increased VEGF is simply the reflection of increased protein leakage in the lung. In clinical studies, increased VEGF in plasma [16], and decreased VEGF in epithelial lining fluid [17], or BAL [18], were noted in ARDS patients. The present study was limited to the first four hours of observation, while these clinical studies were performed within the first couple of days after ARDS developed. It is known that C57BL6 mice are very susceptible to lung hyperoxic stress [41]. These confounding factors may explain the differences between our observation and those of others. Further investigation is required to address these questions.

Despite the increased levels of VEGF in the BAL, a decreased expression of VEGF in the lung tissue, as revealed by the IHC staining, was observed specifically in the IIR group. Factors other than hyperoxia, such as IIR-induced acute inflammatory response, should be responsible for this drop in VEGF. Down-regulation of VEGF has been observed in the rat lungs after four hours of lipopolysaccharide challenge [18]. A down-regulation of VEGF, as well as VEGF receptors, was also found at 24 hours and 72 hours after lipopolysaccharide injection in the mouse lungs [42].

Cell death is a common feature of ALI and ARDS, contributing to the dysfunction of the alveolar-capillary barrier [6]. The role of VEGF as a survival factor for endothelial cells is already well established [43,44]. A correlation between the reduced VEGF levels and endothelial cell death has been found in the lungs of ARDS patients [45]. The function of VEGF in epithelial cells, however, is largely unknown. Recent evidence suggests that VEGF could also be a survival factor for epithelial cells. VEGF stimulated growth of fetal airway epithelial cells [46] and the proliferation of renal epithelial cells [47]. In a rat model of obliterative bronchiolitis, Krebs and colleagues [48] observed that VEGF either directly promoted epithelial regeneration or inhibited epithelial cell death. Tang and co-workers [49] observed that a transient ablation of the gene encoding VEGF in the lung was associated with an increased number of TUNEL-positive cells in the alveolar walls. In the present study, we found a negative correlation between the number of VEGF-positive cells and TUNEL-positive epithelial cells. To further determine the role of VEGF in lung epithelial cell survival, we used siRNA to knock-down VEGF expression in A549 cells. This technique has been successfully used to effectively and specifically reduce the expression of other signal transduction proteins in lung epithelial A549 cells [30] and other cell types [29]. Our data show that the cell viability was significantly reduced by siRNA for VEGF. Therefore, VEGF could be a survival factor for alveolar epithelial cells. On the other hand, these cells are one of the main sources of VEGF in the lung [11]. Thus, the death of alveolar epithelial cells could be partially responsible for the decreased expression of VEGF [50].

VEGFR-1 is normally expressed on epithelial and endothelial cells in the lung [23,51]. Compared with VEGFR-2, the function of VEGFR-1 in the lung is less determined. In the present study, IHC showed a significant decrease in the expression of VEGFR-1 in both the sham and the IIR groups, suggesting that hyperoxia and/or MV may suppress its expression. The decreased expression level of VEGFR-1 was more significant in the IIR group (*p *< 0.01). The significant negative correlation between VEGFR-1 positive cells and epithelial cell death suggests that down-regulation of VEGFR-1 may be due to epithelial cell death. However, when we knocked down the VEGFR-1 protein level with siRNA, the viability A549 of cells was significantly reduced. This observation is intriguing. It suggests that VEGF and VEGFR-1 may modulate survival of lung epithelial cells in an autocrine fashion.

VEGFR-2, mainly located on endothelial cells, but also on epithelial type II cells [23], is responsible for most of the known functions of VEGF in the lung [12] and, in particular, is involved in the anti-apoptotic properties of VEGF on endothelial cells [43]. In the control and sham-operated animals, the strong staining of VEGFR-2 was found mainly on cells located at the corners of the alveolar space with the appearance of type II pneumocytes and alveolar macrophages. In the IIR group, the number of VEGFR-2 positive cells did not change significantly. The morphological features of cells that expressed VEGFR-2, however, suggest that some of them could be the interstitial monocytes. The accumulation of VEGFR-2-positive inflammatory cells was also noted in a model of Bleomycin-induced lung injury [23]. Moreover, in the IIR group, the expression of VEGFR-2 was localized mainly in the cytoplasm, which was not noted in the other two groups. This phenomenon indicates that the function of VEGFR-2 may be altered by the inflammatory responses in the lung tissue. Further investigation with double immunostaining and confocal microscopy may provide more convincing evidence.

When using western blotting and real-time quantitative RT-PCR to measure the protein and mRNA levels of VEGF and its receptors, the results are not statistically significant. The expression and distribution of these molecules are scattered in the lung tissue. When total tissue lysates were used as samples for immunoblotting, or as sources for total RNA extraction, the background from surrounding cells and tissues may mask the changes of VEGF, or its receptors.

## Conclusion

Our results suggest that significant and dynamic changes of expression of VEGF and its receptors take place during IIR-induced acute lung injury. A rapid release of VEGF into alveolar space may occur as a consequence of hyperoxia and/or MV. Released VEGF may partially contribute to the development of non-cardiogenic pulmonary edema. VEGF and VEGFR-1 may play important roles in maintaining alveolar epithelial cell survival. Acute inflammatory responses and alveolar epithelial injury may reduce their expression, which may subsequently lead to cell death. Alternatively, death of alveolar epithelial cells may limit the production of VEGF in the lung. Further investigation with more specific tools or transgenic animals may elucidate the intricate role of VEGF system in acute lung injury.

## Key messages

• During the early stage of ALI induced by IIR, the expression of VEGF and its receptors changes in the lung tissue.

• Increased release of VEGF (found in BLF) may contribute to pulmonary permeability.

• Reduced VEGF and its type I receptor in the lung tissue may affect lung epithelial cell survival.

## Abbreviations

ALI = acute lung injury; ARDS = acute respiratory distress syndrome; BAL = bronchoalveolar lavage; EBD = Evans Blue Dye; FITC = fluorescein isothiocyanate; GAPDH = glyceraldehyde-3-phosphate dehydrogenase; IHC = immunohistochemistry; IIR = intestinal ischemia-reperfusion; MV = mechanical ventilation; PBS = phosphate-buffered saline; RT-PCR = reverse transcriptase PCR; siRNA = small interference RNA; TMR = tetramethylrhodamine; TUNEL = terminal transferase dUTP nick end labeling; VEGF = vascular endothelial growth factor; VEGFR = vascular endothelial growth factor receptor.

## Competing interests

The authors declare that they have no competing interests.

## Authors' contributions

MM designed the study and carried out most of the animal studies, assessment of lung injury and VEGF related molecules in the lung, and drafted the manuscript. BH designed and conducted the *in vitro *siRNA studies, and revised the manuscript. CFA participated in the experimental design and animal studies. RS participated in the assessment of VEGF related molecules and discussion. DH performed lung injury scoring and supervised pathological studies. TKW and SK provided assistance in physiological studies and data interpretation. ML conceived the study, and participated in its design and coordination and finalized the manuscript. All authors read and approved the final manuscript.

## Figures and Tables

**Table 1:** Survival, physiological and lung injury parameters.

	N	Control	Sham	IIR
Survival (percent)	24	NA	100	50^a^
Blood pressure after 4 h (mmHg)	4	NA	51.0 ± 6.1	32.5 ± 10.3^a^
BAL protein concentration (μg/ml)	12	46.1 ± 30.7	149.2 ± 72.0^b^	174.4 ± 82.2^c^
BAL total cell count (× 10^5^/ml)	12	6.8 ± 3.2	11.4 ± 2.1^b^	16.8 ± 7.2^a, c^
BAL neutrophils (percent)	8	4.8 ± 3.6	4.6 ± 3.9	18.5 ± 13.0^d^
EBD permeability assay	4	0.021 ± 0.00	0.024 ± 0.01	0.038 ± 0.00^d^
Compliance percentage of decrease from baseline)	8	NA	0.2 ± 0.0	18.8 ± 8.8^a^

^a^*p *< 0.05 versus sham; ^b^*p *< 0.05 and ^c^*p *< 0.01 versus control; ^d^*p *< 0.05 versus other groups. BAL, bronchoalveolar lavage; EBD, Evans Blue Dye; IIR, intestinal ischemia-reperfusion; NA, not applicable.

**Table 2:** Differential patterns of VEGF and VEGFRs immunostaining in the lung cells.

	VEGF			VEGFR-1			VEGFR-2		
	Control	Sham	IIR	Control	Sham	IIR	Control	Sham	IIR
Bronchial epithelium (ciliated)	+++	+++	++	+++	+++	++	++	++	++
Bronchial epithelium (non-ciliated)	+++	+++	++	++	++	++	++	++	++
Type I cells	+	+	-	++	+	+	-	-	-
Type II cells	++	++	+	+++	++	+	+	++	++
Interstitial cells	-	-	-	++	+	+	-	-	-
Vascular endothelium	++	++	++	+++	+++	++	+	+	+
Alveolar macrophages	+	+	+	++	+	+	+	+	+

Unstained, -; occasional staining, +; weak to moderate diffuse staining, ++; strong diffuse staining, +++. IIR, intestinal ischemia-reperfusion; VEGF, vascular endothelial growth factor; VEGFR, vascular endothelial growth factor receptor.

**Table 3:** Correlation of VEGF levels with protein concentrations and cell counts in bronchoalveolar lavage fluid.

	n	*Rho*	*p*
Protein (μg/ml)	30	0.79	<0.0001
Total cells (× 10^5^/ml)	30	0.62	0.0002
Macrophages (percent)	9	0.73	0.028
Macrophages (× 10^5^/ml)	9	0.50	0.260
Neutrophils (percent)	9	-0.83	0.007
Neutrophils (× 10^5^/ml)	9	-0.79	0.036
